# Call to action: Accrediting body for graduate veterinary medical education

**DOI:** 10.1111/jvim.16867

**Published:** 2023-11-17

**Authors:** Jane E. Sykes

**Affiliations:** ^1^ University of California‐Davis Davis California USA

Dear Editors,

In response to growing concerns from the membership of the American College of Veterinary Internal Medicine (ACVIM) regarding several interrelated complex issues facing the veterinary specialty profession, in April of 2023, the ACVIM Board of Regents (BOR) drafted a letter to the American Veterinary Medical Association (AVMA) requesting that the AVMA convene a group of stakeholders to explore the formation of an overarching accreditation body for veterinary graduate clinical education. It was proposed that the accreditation body be named the Accreditation Council for Graduate Veterinary Medical Education (ACGVME) for consistency with the Accreditation Council for Graduate Medical Education (ACGME). The letter was subsequently shared with the American Animal Hospital Association (AAHA), the American Association of Veterinary Clinicians (AAVC), the American Association of Veterinary Medical Colleges (AAVMC), the newly formed Veterinary Specialty Coalition (VSC) group, and the Veterinary Specialty Organizations Committee of the AVMA (VSOC). Co‐signatures were obtained from AAHA, AAVMC, the American College of Veterinary Emergency and Critical Care (ACVECC), and the Competency‐Based Veterinary Specialty Education Taskforce of the AAVMC. Leadership of the AAVC also indicated their support for the request, noting they had already written to the AVMA requesting similar action. On July 14, 2023, the letter was posted on ACVIM360 and the BOR considered input from the ACVIM membership before delivering the letter to the AVMA on August 15, 2023.

The ACGME is a nonprofit private council that is responsible for evaluating and accrediting all graduate medical training programs (internships, residencies, and fellowships) for physicians in the United States. The evolution of the ACGME is described in an accompanying perspectives article in this issue of the *Journal of Veterinary Internal Medicine*.^1^ The following represents an excerpt of the letter to the AVMA.

While individual specialty colleges (such as ACVIM), private specialty practices, and colleges of veterinary medicine have established mechanisms to oversee training program quality, there is no consistency, coordination, or oversight in this effort. Although the AVMA and AAVMC have developed guidelines for veterinary internships, there is no one body to ensure that training institutions adhere to standards. The lack of oversight within the veterinary profession is astonishing, given that standards for internship programs were set by the AMA Council on Medical Education in 1914, and an Internship Review Committee was formed in 1954 (with representatives from the AMA, American Association of Medical Colleges, American Hospital Association, and Federation of State Medical Boards).

There is an urgent need for a single organization to address the following growing and interconnected problems in veterinary specialty medicine:

1. *Lack of broad oversight to ensure consistency in the quality of intern, resident, and fellowship training programs*: Program registration and review processes differ among specialty colleges, and some specialty colleges have required training institutions to pay a residency training program (RTP) fee to support the costs of RTP oversight. Such fees have been controversial, and no one body is working to ensure such fees are reasonable.
2. *A crisis relating to intern and resident wellbeing*: This crisis resulted in the development of the Guidelines for Veterinary Intern & Resident Wellbeing by the American Association of Veterinary Medical Colleges; a national meeting on the topic; and efforts to encourage corporate veterinary practices, academia, and specialty colleges to adopt the Guidelines.
3. *A crisis of diversity, equity, and inclusion*: A lack of diversity among veterinary specialists exists and many veterinary specialty colleges, including our college, are expending significant resources to understand the problem and work to be more inclusive, but more support is required. The AAVMC has made great strides with their DiVersity Matters initiative, but their efforts are not focused on the veterinary specialty community as a whole. Building diverse physician workforce is a key component of the ACGME's mission and the ACGME has detailed Common Program Requirements that address issues of diversity, equity, and inclusion for internships, residencies, and fellowships.
4. *A national shortage of veterinary specialists*: This stimulated the recent national Specialty Veterinary Medicine Stakeholder Summit that was attended by 33 stakeholders from privately held and corporate‐owned practices, academia, industry, and specialty colleges.
5. *A mismatch between available internship graduates and residency training programs (RTPs)*: Some practices that offer quality residency training programs (RTPs) face an insufficient pool of intern applicants (or complete lack of applicants). Yet every year some of the brightest and most talented intern graduates are not offered positions, despite the dire shortage of veterinary specialists.
6. *Proliferation of veterinary specialty internships*: Over the last decade, there has been a proliferation of “specialty” internship training programs in both academia and private practice. Graduating interns enter these programs because they fail to match to an RTP after completing a traditional internship; or because they choose to delay residency training for financial or personal considerations (wellbeing, family pressures). Thus, graduates from traditional internship programs now often compete with graduates from “specialty” internship training programs (eg, surgery internships, medicine internships) for open positions in attractive RTPs. When new graduates learn that they may need to complete a traditional internship then a second “specialty” internship to be selected into top RTPs they are further deterred from pursuing a career as a specialist.

It is not surprising that many new veterinary graduates choose not to pursue a career in specialty medicine because they face (1) insufficient salaries to repay educational debt and/or support basic costs of living; (2) excessive working hours; (3) poor quality training programs that lack structure, an appropriate caseload, equipment/facilities, support staff, research and educational opportunities; and (4) inadequate mentorship and supervision.

The AAVC, ACVIM, AAHA, ACVECC, and the competency‐based veterinary specialty education taskforce have now urged the AVMA to engage stakeholders from academia, organized veterinary medicine, corporate practices, and industry to begin discussions regarding the formation and activities of the new organization, the Accreditation Council for Graduate Veterinary Medical Education (ACGVME), for consistency with the Accreditation Council of Graduate Medical Education (ACGME). Members of the ACVIM Board of Regents look forward to participating in these discussions for the benefit of the specialties of Small Animal Internal Medicine, Large Animal Internal Medicine, Cardiology, Neurology, Nutrition, and Oncology, as well as the veterinary profession and the animal‐owning public.

## CONFLICT OF INTEREST DECLARATION

The author is Chair of the Board of Regents of the American College of Veterinary Internal Medicine, the society owner of the Journal of Veterinary Internal Medicine. The author was not involved in the review of this article or the decision to publish it.
